# Combined effect of traditional Chinese herbal-based formulations Jing Si herbal tea and Jing Si nasal drop inhibits adhesion and transmission of SARS-CoV2 in diabetic SKH-1 mice

**DOI:** 10.3389/fphar.2022.953438

**Published:** 2022-11-08

**Authors:** Chien-Yi Chiang, Wei-Wen Kuo, Yu-Jung Lin, Chia-Hua Kuo, Cheng-Yen Shih, Pi-Yu Lin, Shinn-Zong Lin, Tsung-Jung Ho, Chih-Yang Huang, Marthandam Asokan Shibu

**Affiliations:** ^1^ Cardiovascular and Mitochondrial Related Disease Research Center, Hualien Tzu Chi Hospital, Buddhist Tzu Chi Medical Foundation, Hualien, Taiwan; ^2^ Jing Si Herbal Research and Application Center, Hualien, Taiwan; ^3^ Department of Biological Science and Technology, College of Life Sciences, China Medical University, Taichung, Taiwan; ^4^ Ph.D. Program for Biotechnology Industry, China Medical University, Taichung, Taiwan; ^5^ Laboratory of Exercise Biochemistry, University of Taipei, Taipei, Taiwan; ^6^ Buddhist Tzu Chi Charity Foundation, Hualien, Taiwan; ^7^ Buddhist Tzu Chi Bioinnovation Center, Buddhist Tzu Chi Medical Foundation, Hualien, Taiwan; ^8^ Department of Neurosurgery, Hualien Tzu Chi Hospital, Hualien, Taiwan; ^9^ Department of Chinese Medicine, Hualien Tzu Chi Hospital, Buddhist Tzu Chi Medical Foundation, Hualien, Taiwan; ^10^ Integration Center of Traditional Chinese and Modern Medicine, Hualien Tzu Chi Hospital, Hualien, Taiwan; ^11^ School of Post-Baccalaureate Chinese Medicine, College of Medicine, Tzu Chi University, Hualien, Taiwan; ^12^ Department of Medical Laboratory Science and Biotechnology, Asia University, Taichung, Taiwan; ^13^ Graduate Institute of Biomedical Sciences, China Medical University, Taichung, Taiwan; ^14^ Center of General Education, Buddhist Tzu Chi Medical Foundation, Tzu Chi University of Science and Technology, Hualien, Taiwan; ^15^ Department of Medical Research, China Medical University Hospital, China Medical University, Taichung, Taiwan; ^16^ Department of Biotechnology, Bharathiar University, Coimbatore, India

**Keywords:** herbal formula, SARS-CoV2 variants, diabetes, viral transmission, phytotherapy, *Artemisia argyi*, *Ohwia caudata*

## Abstract

Multiple studies show increased severity of SARS-CoV2-infection in patients with comorbidities such as hypertension and diabetes. In this study, we have prepared two herbal-based formulations, a pleiotropic herbal drink (Jin Si Herbal Tea, JHT) and a nasal drop (Jin Si nasal drop, JND), to provide preventive care against SARS-CoV2 infection. The effect of JHT and JND was determined in SARS-CoV2-S-pseudotyped lentivirus-infected bronchial and colorectal cell lines and in SKH-1 mouse models. For preliminary studies, ACE2 receptor abundant bronchial (Calu-3) and colorectal cells (Caco-2) were used to determine the effect of JHT and JND on the host entry of various variants of SARS-CoV2-S-pseudotyped lentivirus. A series of experiments were performed to understand the infection rate in SKH-1 mice (6 weeks old, n = 9), find the effective dosage of JHT and JND, and determine the combination effect of JHT and JND on the entry and adhesion of various variant SARS-CoV2-S-pseudotyped lentiviruses, which included highly transmissible delta and gamma mutants. Furthermore, the effect of combined JHT and JND was determined on diabetes-induced SKH-1 mice against the comorbidity-associated intense viral entry and accumulation. In addition, the effect of combined JHT and JND administration on viral transmission from infected SKH-1 mice to uninfected cage mate mice was determined. The results showed that both JHT and JND were effective in alleviating the viral entry and accumulation in the thorax and the abdominal area. While JHT showed a dose-dependent decrease in the viral load, JND showed early inhibition of viral entry from day 1 of the infection. Combined administration of 48.66 mg of JHT and 20 µL of JND showed rapid reduction in the viral entry and reduced the viral load (97–99%) in the infected mice within 3 days of treatment. Moreover, 16.22 mg of JHT and 20 µL JND reduced the viral infection in STZ-induced diabetic SKH-1 mice. Interestingly, combined JHT and JND also inhibited viral transmission among cage mates. The results, therefore, showed that combined administration of JHT and JND is a novel and an efficient strategy to potentially prevent SARS-CoV2 infection.

## Introduction

The COVID-19 pneumonia pandemic is considered in many regions to be on the verge of becoming endemic, but there is still no specific drug available for prevention and its treatment. The virus has already infected over 220 million people and has caused 4.5 million recorded deaths worldwide (Jin and Hu 2021). The available therapeutics show inconclusive efficiency, and as the virus develops various mutations and spreads as stronger variants, the need to explore other avenues beyond vaccines has become paramount (Dowarah et al., 2021; GloPID-R, UKCDR, and COVID-19 Clinical Research Coalition Cross-Working Group on COVID-19 Research in LMICs, 2021). Novel therapeutic strategies without concerns on universal availability will provide wider coverage and help the population not appropriate for available immunization. As the mechanism of SARS-CoV2 infection is now well-elucidated, it is now possible to screen therapeutic drugs to target and prevent the viral infection. While laboratory studies on SARS-CoV2 infection require BSL-3 or BSL-4 containment facilities, pseudotyping of glycoproteins from SARS-CoV2 viruses onto low-risk group viruses that only require a BSL-2 facility has provided a viable method to carry out high-throughput screening of entry inhibitors for viruses (Whitt 2010).

Recent studies show that the binding of spike (S) protein of SARS-CoV2 with host receptors like ACE2, dipeptidyl peptidase 4 (DPP4), and aminopeptidase-N (APN) facilitate the viral entry (Shatizadeh Malekshahi et al.; Valencia et al., 2020). In this context, it should, however, be noted that ACE2 expression is an important player in protecting the lung against injury, and ACE2 expression has, in fact, been shown to decrease in SARS-CoV2 infection (Hoffmann et al., 2020; Lan et al., 2020; Wan et al., 2020; Nicosia et al., 2021).

Upon adhesion to the receptor ACE2, the SARS-CoV2 hijacks the cellular serine protease machinery to initiate its entry (Simmons et al., 2005). Proteases like TMPRSS2 found in ACE2-positive cells help SARS-CoV2 priming to the host by a process similar to activation and host entry of other coronaviruses and influenza viruses (Matsuyama et al., 2010; Glowacka et al., 2011). Interventions that block this specific mechanism of viral entry could effectively prevent viral entry and provide a strategy to reduce the susceptibility to SARS-CoV2-S infection (Senapati et al., 2021).

Older adults and people of any age who have underlying medical conditions, such as hypertension and diabetes, have shown worse prognosis. Diabetic patients have increased morbidity and mortality rates and have been linked to more hospitalization and intensive care unit admissions. With the available data, it has been observed that comorbidities such as diabetes increase the chances of infection and mortality (Sanyaolu et al., 2020). Therefore, interventions that provide better protection against SARS-CoV2-S infection in high-risk patients will help in reducing the morbidity and mortality rates and traditional Chinese medicine practices have identified various such herbal candidates.

In our study, to find herbal supplements against SARS-CoV2-S infection, a Chinese medicinal herbal-based drink (Jin Si Herbal Tea, JHT) and a nasal drop (Jin Si nasal drop, JND) were formulated to complement the conventional therapy for COVID-19 patients. The constituents of JHT and JND have been previously investigated and have shown promising evidence for antiviral effects and protection against comorbidities, which forms the rationale for the antiviral preparation. The constituents of JHT that include leaves of *Artemisia argyi* H.Lév. & Vaniot [Family *Asteraceae*] (also called “Chinese mugwort”), *Ohwia caudata* (Thunb.) H.Ohashi [Family *Fabaceae*], *Ophiopogon japonicas* (Thunb.) Ker Gawl. [Family Asparagaceae], *roots of Houttuynia cordata* Thunb*.* [Family *Saururaceae*], *Platycodon grandifloras* (Jacq.) A.DC. [Family *Campanulaceae, Glycyrrhiza uralensis* Fisch. ex DC. [Family *Fabaceae*, *Radix Glycyrrhizae*], *Perilla frutescens* (L.) Britton [Family Perilla], and flowers of *Chrysanthemum × morifolium* (Ramat.) Hemsl [Family *Asteraceae*] have shown potential antiviral or anti-inflammatory effects (Lau et al., 2008; Zhang et al., 2016; Liu et al., 2020a; de Almeida-Pititto et al., 2020; Kwon et al., 2020; Mahrosh and Mustafa 2021; Kim et al., 2021; Tang et al., 2021). Recent findings suggest that JHT possesses anti-diabetic effects and overall rejuvenating effects in various models (Shibu et al., 2022). The constituents of JND include *Zingiber officinale* Roscoe [Family *Zingiberaceae*] or old ginger, *Smilax glabra* Roxb*.* [Family *Smilacaceae*], *Artemisia argyi* H.Lév. & Vaniot*, Ohwia caudata* (Thunb.) H. Ohashi*, Perilla frutescens* (L.) Britton and *Mentha canadensis* L. (Family *Lamiaceae,* menthe).

In our attempt to find a traditional Chinese medicine strategy, we examined the individual and combined administration of oral JHT and nasal JND formula as prevention for SARS-CoV2-S infection in SKH-1 mouse models by analyzing the adhesion and transmission of SARS-CoV2-S-pseudotyped lentiviruses.

## Methods and materials

### Materials

All the chemicals were purchased from Sigma-Aldrich, St. Louise, MO, United States). The plant materials used were identified by an experienced botanist (Dr. Tamilselvi Shanmugam, China Medical University Hospital, Taiwan) and Prof. Tsung-Jung Ho with reference to the Taiwan Herbal Pharmacopoeia 2018, Chinese Version III, and the species were fully validated with the medicinal plant service of Royal Botanic Gardens Kew Science. The plant materials used are maintained in the herbarium of Hualien Tzu Chi Hospital, Taiwan. The wild-type, alpha, delta, epsilon, and gamma variants of pseudotyped viral particles were obtained from RNAicore, Academia Sinica, Taiwan. The viral particles were prepared as reported earlier (Lee et al., 2022). Briefly, the pseudotyped viral particles were obtained by co-transfecting Lenti-X 293T cells with transfer plasmid (pLAS2w.Nluc-T2A-RFP-C.Ppuro, 5 μg), packaging plasmid (pCMVdeltaR8.91, 4 μg), and 1 μg spike-expressing plasmid (wild type, alpha, delta, epsilon, and gamma variants of pcDNA3.1-2019-nCoV-S-d18 in a 10-cm culture dish. Supernatants collected after 30 and 60 h of transfection were obtained and stored as viral stocks.

### Jing Si herbal tea

The constituents of JHT include leaves of *Artemisia argyi* H.Lév. & Vaniot, *Ohwia caudata* (Thunb.) H.Ohashi, *Ophiopogon japonicas* (Thunb.) Ker Gawl., *roots of Houttuynia cordata* Thunb, *Platycodon grandifloras* (Jacq.) A.DC., *Glycyrrhiza uralensis* Fisch. ex DC., *Perilla frutescens* (L.) Britton, and flowers of *Chrysanthemum × morifolium* (Ramat.) Hemsl. The materials were washed, and 6 g of *A. argyi*, 6 g of *Ohwia caudata*, 4 g of *Ophiopogon japonicus*, 4 g of *H. cordata*, 4 g of *Platycodon grandiflorus*, 2 g of *G. uralensis*, 2 g of *Perilla frutescens*, and 0.2 g of *Chrysanthemum ×morifolium* were mixed and finely powdered and added to 600 ml and concentrated to 60 ml by boiling. The preparation was spun down (slow speed) to remove the pellet and filtered through a 0.45-µm filter and characterized by LC/MS.

### Jing Si nasal drop

In 675 ml of autoclaved RO water old ginger (7.5 g), *S. glabra* Roxb*.* (22.5 g), *A. argyi* H.Lév. & Vaniot (22.5 g), *O. caudata* (Thunb.) H.Ohashi (22.5 g), *P. frutescens* (L.) Britton (22.5 g), and menthe (22.5 g) were boiled for 30 min. The contents were filtered, and to 100 ml (30 mg/ml) of the collected extract, 600 ml of rice wine and 300 ml of 95% alcohol were added, mixed well, and filtered. To the filtrate, 6 ml of tea tree oil was added and mixed well and collected as the final preparation of JND. The preparation was characterized by LC-MS analysis.

### LC-MS analysis

After filtering through a 0.45-µm filter, 100 μL of each sample was mixed with 200 uL of methanol and shaken well, and the solution was left at -20°C for 30 min, centrifuged at 15,000 x g for 10 min, and the supernatant was collected after 1 h and used for analysis. The chromatographic separation and mass spectrometric detection of samples were performed using Triple Quad (LC-MS/MS) (Waters Acquity, Milford, MA, United States), and on a C18 column (150 mm length). Injection volume was 5 μL, and the mobile phase was a mixture of A: 0.1% (v/v) formic acid/water; B: 0.1% (v/v) formic acid/acetonitrile. The flow rate was 100 μL/min, and the elution was performed starting from 95% A to 5% B, and a gradient increase of B and a gradient elution program were used. Both positive and negative spectrums were obtained and fraction peaks of JHT (Supplementary Figure S1) and JND (Supplementary Figure S2) were characterized with respective spectrums. Furthermore, the presence of swertisin, isoliquiritigenin, and eupatilin in JHT (Supplementary Figure S3) and JND (Supplementary Figure S4) was characterized based on the chromatograms of standard chemicals (Shibu et al., 2022). The concentration of swertisin, isoliquiritigenin, and eupatilin in JHT was determined as 21720 ± 1640 ng/ml, 1504 ± 72 ng/ml, and 2252 ± 20 ng/ml, respectively. Similarly, the concentrations of swertisin, isoliquiritigenin, and eupatilin in JND were 24,280 ± 440 ng/ml, 0.9040 ± 0.120 ng/ml, and 241 ± 7 ng/ml, respectively.

### Cell culture

Human lung grade I adenocarcinoma Calu-3 cells (ATCC HTB-55, P8-P10) and colorectal Caco-2 (ATCC: HTB-37, P8-P10) cells were cultured in Eagles Minimum Essential Medium containing 20% fetal bovine serum (Gibco, MA, United States), and H9c2 cells (BCRC, Taiwan) were maintained in DMEM supplemented with 20% fetal bovine serum. The cells were cultured in a CO_2_ incubator maintained at 37°C with 95% air and 5% CO_2_. To check the cytotoxicity effects of JHT and JND, their effect on cell viability was measured using H9c2 cardiomyoblasts and Calu-3 (lung) cells by MTT [3-(4,5- dimethylthiazol-2-yl)-2,5-diphenyltetrazolium bromide] assay (Liu et al., 2020b; Shibu et al., 2022). For the MTT assay, 2 × 10^3^ cells were treated with different concentrations of JHT and JND for 24 h. To determine the inhibition of viral adhesion by JHT and JND, a viral infection assay was performed on either Calu-3 cells or Caco-2 cells with fluorescent SARS-CoV-2-S protein pseudo-type lentiviruses (10 MOI). JHT (100 mg/ml), and JND (35 mg/ml) were added to 60% confluent Calu-3 or Caco-2 cells, cultured in 10 ml of media, by replacing 1, 10, and 100 µL media with appropriate volume, and the controls were added with 100 µL of the mixture containing rice wine (17–20% alcohol, Taiwan Tobacco and Liquor Corporation, Taiwan), ethanol, ddH_2_O, and tea tree oil (600:300:100:6). After 2 hours, the viral particles were added to the culture, and the results were observed using a fluorescence microscope after 48 h.

### Animal experiments

This study was conducted following the protocol and approved by the Institutional Animal Care and Use Committee (IACUC) of Hualien Tzu Chi Hospital, Taiwan. Two weeks prior to the experiments, the mice (6-week-old male C57BL/6 and SKH-1) were allowed to adapt to the environment and the diet. During the 2-week adaptation period, all the animals were housed in a room maintained at 24 ± 2°C and 55 ± 10% humidity with a 12-h light cycle. The animals were fed with a standard laboratory diet (PMI Nutrition International, Brentwood, MO, United States) and were provided with reverse osmosis-treated water *ad libitum*.

Eight-week-old SKH-1 mice were used to ascertain the effect of JHT on viral adhesion and infective viral load. The treatment mice were pre-treated with 300 µL of JST (48.66 mg/mice/day) and two doses of JND for 3 days. Two hours after the second dosage of JND, SARS-CoV2 was nasally administered with 500 μL of 1.2 × 10^6^ luminescence viral particles every day for three continuous days using a nebulizer (Aeroneb USB controller, Kent Scientific Corporation Torrington, CT, United States) with a flow rate of 0.4 ml/min. On the fifth day, the viral load accumulated in the mice was determined by imaging using an *In Vivo* Imaging System (IVIS, PerkinElmer, United Kingdom). Nine mice were used in each group.

For diabetes induction, one shot of STZ (60/mg/kg, IP) was administered, and the mice were considered diabetic if their fasting glucose levels remained >200 mg/dl within 3 days of STZ injection as detected by Accu Soft (Hoffmann-La Roche) test strips. From the following day, the JHT/JND treatment and the viral challenge were given every day for 3 days using oral gavage.

### Viral transmission assays

The viral transmission assay was performed based on the transmission of the virus from viral-infused mice to un-infused cage mate mice. In a cage containing three mice, a susceptible control, a viral challenged mouse, and one treated with oral JHT and nasal JND. After 1 and 2 days of viral challenge, the unchallenged mice were analyzed by IVIS to quantify the possible transmitted viral accumulation.

## Results

### Cytotoxic evaluation of JHT and JND by MTT assay

**FIGURE 1:**
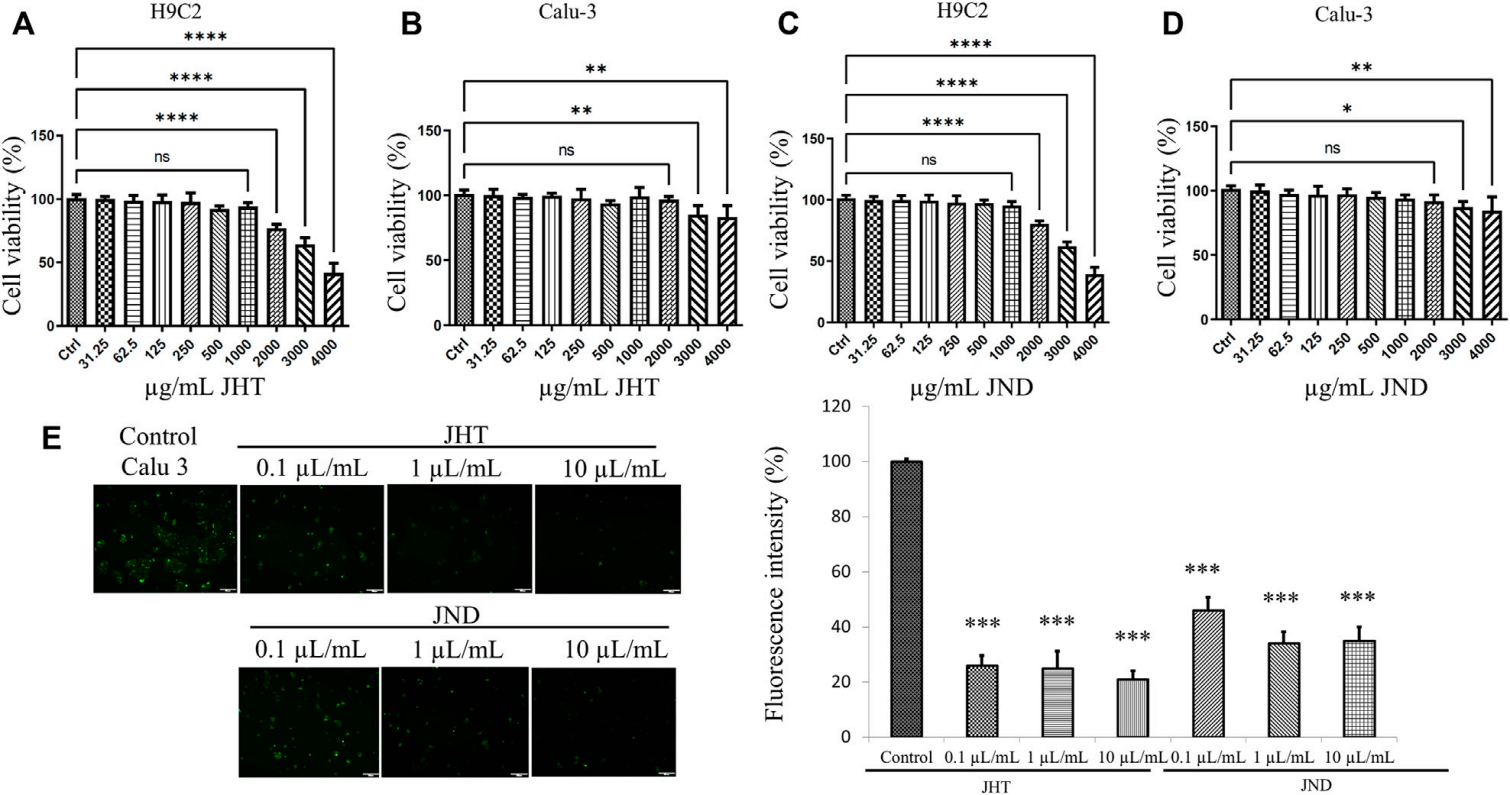
Cytotoxic evaluation of JHT and JND and their effects on SARS-CoV2-S-pseudotyped lentivirus penetration in ACE receptor-rich Calu-3 cells. Cytotoxicity of JHT **(A**,**B)** and JND **(C**,**D)** was evaluated by MTT assay in H9c2 **(A**,**C)** cells and in Calu-3 cells **(B**,**D)**. Calu-3 cells infected with alpha (B.1.1.7) SARS-CoV2-S-pseudotyped lentivirus display green fluorescence with or without 1 μL, 10 μL, and 100 µL of JHT or JND **(E)**. The fluorescence intensity was measured with respect to the number of cells in four fields of view from fluorescence microscope images. The experiments were repeated thrice, and values represent mean ± SD. *** = *p* < 0.001 represents significant difference when compared with control Calu-3 cells.

Cytotoxic effects of JHT and JND were determined by the MTT assay in two different cell lines—an embryonic-derived cardiomyoblast cell line H9c2 and a human lung grade I-adenocarcinoma Calu-3 cell line. The results showed that the JHT (Figures 1A,B) and JND (Figures 1C,D) do not cause any toxic effect in the *in vitro* studies up to 1000 μg/ml in both H9c2 cells (Figures 1A,C) and Calu-3 cells (Figures 1B,D). However, concentrations over 1000 μg/ml showed a dose-dependent cytotoxic effect in both JHT and JND.

### JHT and JND inhibit SARS-CoV2-S-pseudotyped lentivirus infection in ACE receptor-rich bronchial cells (Calu-3) and Caco-2 cells

**FIGURE 2:**
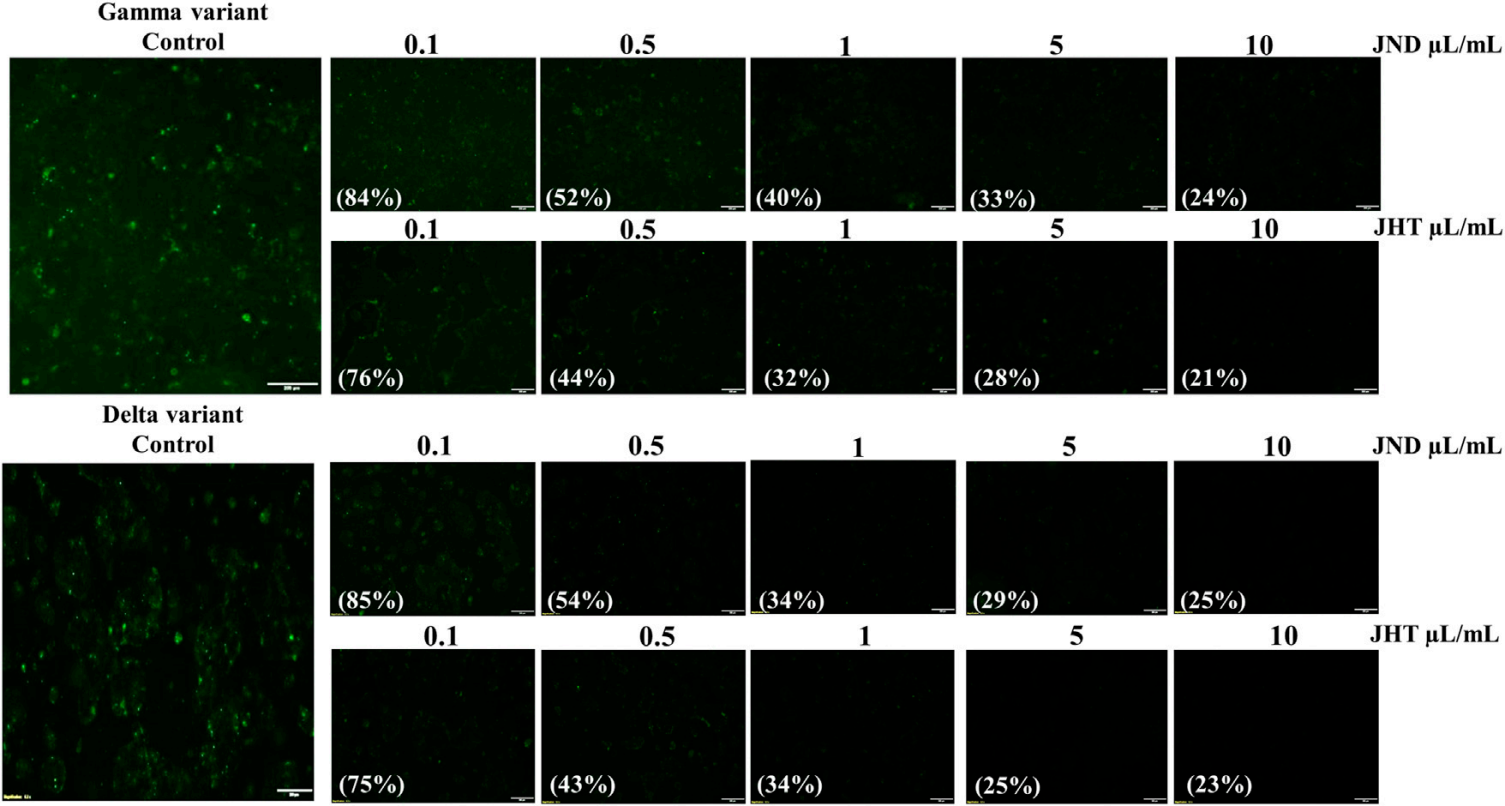
Effect of JHT and JND on gamma and delta variants of SARS-CoV2-S-pseudotyped lentivirus with high transmission rates: Caco-2 cells infected with delta and the gamma mutants with or without 0.1 μL/ml, 0.5 μL/ml, 1 μL/ml, 5 μL/ml, and 10 μL/ml of JHT or JND.

Calu-3 cells infected with alpha (B.1.1.7) SARS-CoV2-S-pseudotyped lentivirus showed green fluorescence, indicating the ability for viral adhesion and entry of the viral RNA. However, Calu-3 cells pretreated with 0.001–0.1 ml of JHT and JND showed a reduction in the green fluorescence, showing the effect of JHT and JND in inhibiting the viral entry (Figure 1E). Similarly, the effect of JHT and JND on variants seen to be with high transmission rates and gastrointestinal infection such as the delta and the gamma mutants was identified. The results show that the delta and gamma variants of pseudotyped viruses on Caco-2 cells showed green fluorescence, but pretreatment with JHT and JND showed a reduction in green fluorescence, indicating the inhibition of viral entry in the colorectal cells (Figure 2).

### Combined effect of JHT and JND is rapid and effective in reducing viral load in the thorax and in the abdomen

**FIGURE 3:**
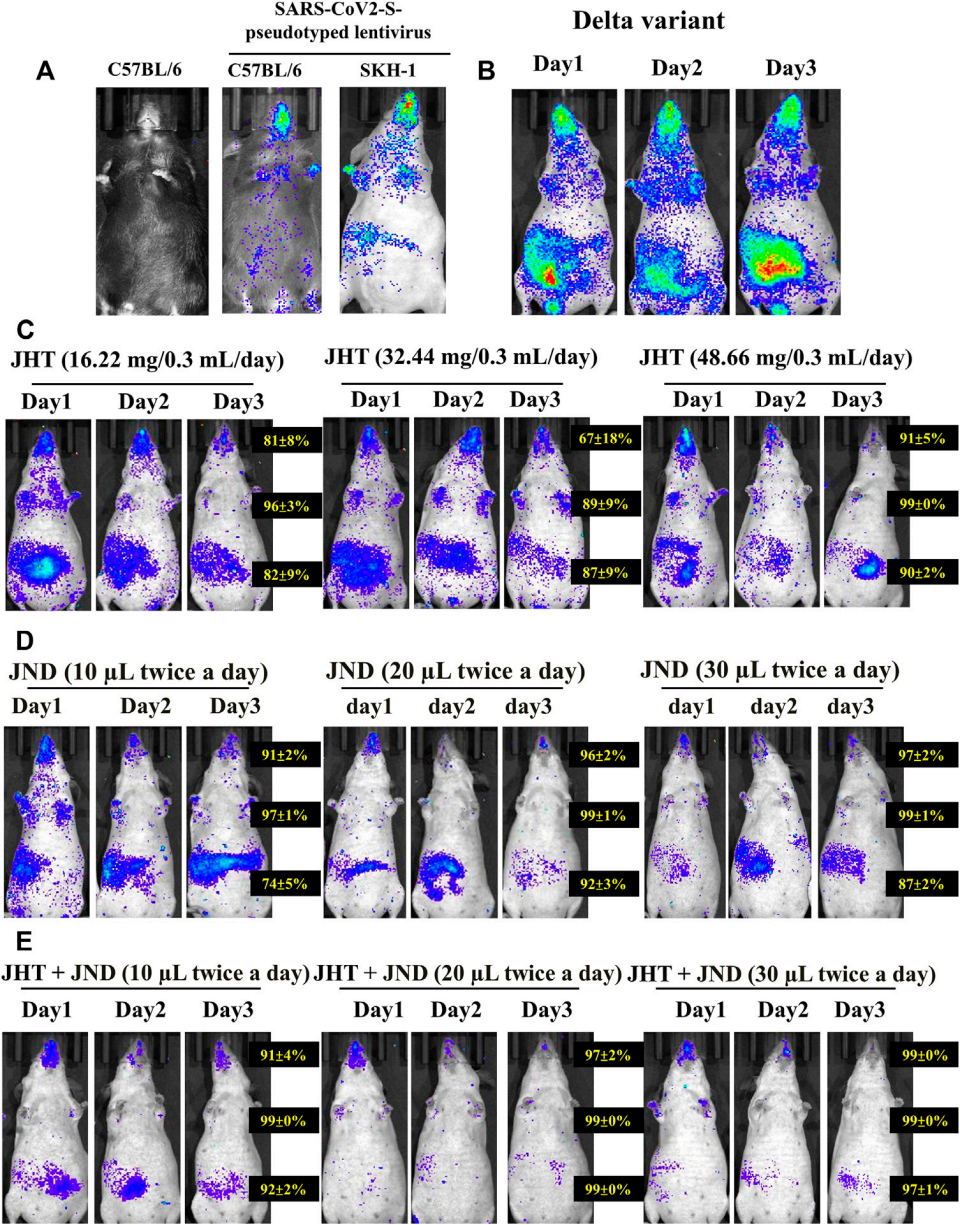
Effect of combined oral JHT and JND in reducing viral load in the thorax and in the abdomen. **(A)** Viral infusion shows variation in infection between C57BL/6 strains and SKH-1 strain of mice (n = 9) as seen from IVIS. **(B)** Infection with delta variants infused on Day 0 and continued infusion for the next 3 consecutive days. Time-dependent (1 day, 2 days, and 3 days) and a dose-dependent analysis with 16.22 mg, 32.44 mg, and 48.66 mg of JHT **(C)** and 10 μL, 20 μL, and 30 µL JND **(D)** and the effect of combined 48.66 mg JHT and different doses of JND **(E)** on the viral load in SKH-1 mice.

Analysis with IVIS 1 day after challenge with alpha variants showed infection in the thorax and the abdomen of SKH-1 cells more efficiently than in C57BL/6 mice (Figure 3A) as seen from the fluorescent intensity in the IVIS images. Interestingly, the delta variant showed much higher entry as evidenced from the heavy viral load in the oral-nasal cavity, thorax, and abdomen. Previous reports performed with SARS-CoV2 wild-type viruses and associated pseudotyped viruses showed that alpha, beta, and gamma variants were successful in entering into C57BL/6 mice, but the delta variants were not (Shuai et al., 2021). This was correlated with the lack of mutation N501Y in wild-type viruses and the delta variants (Shuai et al., 2021; Zhan et al., 2022). However, our studies with SARS-CoV2-S-pseudotyped alpha lentivirus showed very low infection in C57BL/6 compared to SKH-1, and the delta variant also showed very high entry in SKH-1 mice. Therefore, SKH-1 is a better animal model than the C57BL/6 for SARS-CoV2-S-pseudotyped lentivirus infection studies.

Three days of infusion, however, did not show any time-dependent increase or any dependence of repetition on the viral load (Figure 3B). A time-dependent and a dose-dependent analysis on the viral load in the abdomen and the thorax show that treatment with 16.22 mg, 32.44 mg, and 48.66 mg of JHT provides a dose- and time-dependent inhibition in the viral load of delta variants both in the thorax and in the abdomen (Figure 3C). Comparative analysis of 1 day to 3 days of treatment shows that mice with 3 days of JHT treatment were more effective than the short-term treatment groups. Moreover, 10 μL, 20 μL, and 30 µL of JND treatment groups showed better dose dependence in reducing the viral load in the pseudotyped lentivirus thorax (Figure 3D). Although the viral load in the abdomen was reduced in a dose-dependent effect, their effect did not reduce the gut viral load with prolonged treatment. Interestingly, combined treatment with 48.66 mg of JHT and different doses of JND for 3 days showed an effective reduction in the viral load in both the abdomen and thorax within 1 day of treatment. A combination of 10 µL of JND with 48.66 mg of JHT showed a 91% reduction in the nasopharyngeal accumulation, 99% reduction in the thorax, and 92% accumulation in the gut (Figure 3E). Three days of treatment with 30 µL of JND showed a 99% reduction in the nasopharyngeal accumulation, 99% reduction in the thorax, and 97% accumulation in the gut. Therefore, oral JHT and nasal JND can effectively inhibit the viral entry and accumulation systemically.

### JHT and JND effectively inhibit viral infection by various variants of SARS-CoV2-S-pseudotyped lentivirus in both the abdomen and the thorax

**FIGURE 4:**
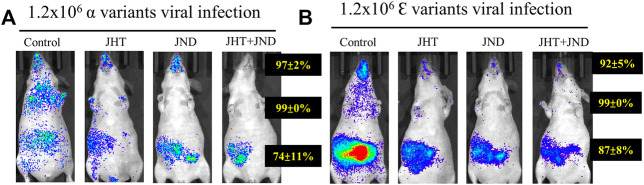
Effect of oral JHT and nasal JND administration on the accumulation of other variants in SKH-2 mice. Alpha **(A)** and epsilon **(B)** variants of SARS-CoV2-S-pseudotyped lentivirus infused with or without oral JHT (48.66 mg in 0.3 ml/day) or nasal JND (30 µL twice a day) administration or combined JHT (48.66 mg in 0.3 ml/day) and JND (30 µL twice a day) with viral infusion, SKH-1-susceptible control, SKH-1 with viral infusion, and JHT and JND treatment mice (n = 9).

Treatment with both JHT and JND effectively reduced the viral load of other variants of SARS-CoV2-S-pseudotyped lentivirus. Oral treatment with 48.66 mg in 0.3 ml/day of JHT or nasal administration of 30 µL of JND reduced the infection of SARS-CoV2-S- α-pseudotyped lentivirus (α variant, Figure 4A) and SARS-CoV2-S- Ɛ -pseudotyped lentivirus (Ɛ variant, Figure 4B) in the thorax and in the abdomen. Combined treatment with JHT and JND showed comparably a better reduction in the viral load in the abdomen as seen from the reduced fluorescent intensity in the IVIS images (Figures 4A,B).

### JHT inhibits viral accumulation in diabetic SKH-1 mice and the combined effect of JHT and JND inhibits transmission of SARS-CoV2-S- delta variant pseudotyped lentivirus to unchallenged groups

**FIGURE 5:**
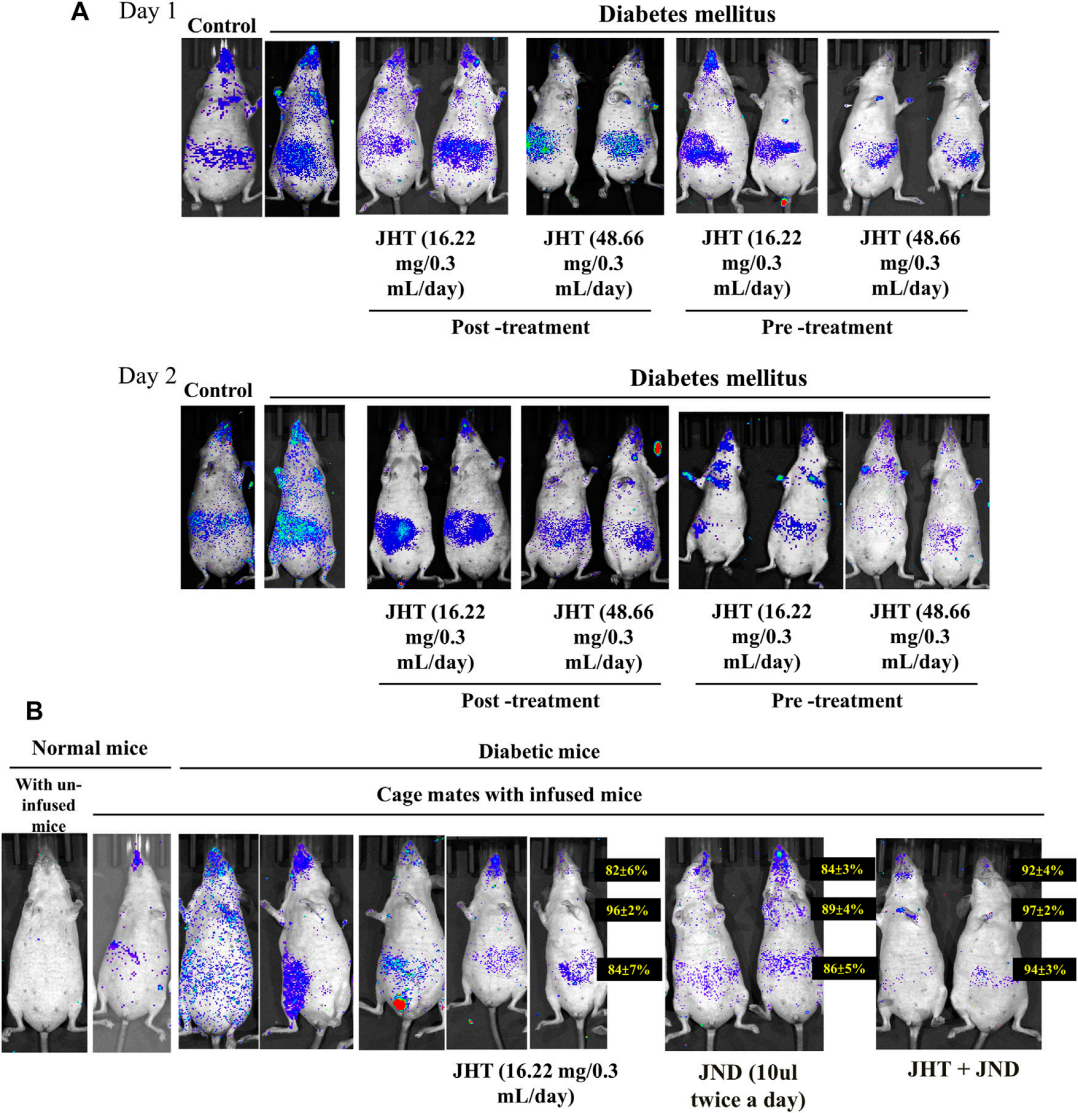
Effect of JHT and JND on the viral accumulation in diabetic SKH-1 mice and combined effect of JHT and JND on the transmission of SARS-CoV2-S- gamma variant: Viral load in diabetic SKH-1 mice (n = 9) with or without 16.22 mg and 48.66 mg post-treatment/pre-treatment of JHT administration for 2 days **(A)**. Analysis of the viral transmission within cage mates shows viral load in mice infused with SARS-CoV2-S- gamma variant pseudotyped lentivirus and in those not infused with/without JHT, JND, and combined JHT and JND administration **(B)**.

Infusion of nasal SARS-CoV2-S-gamma variant pseudotyped lentivirus in STZ-induced diabetic SKH-1 mice showed an increase in the viral load compared to the normal SKH-1 mice. Post-treatment with 16.22 mg and 48.66 mg of JHT after 2 days of viral infusion showed effective reduction in the viral load in the thorax and a slight reduction in the gut. However, JHT pretreatment showed effective reduction in the thorax and the abdomen viral load upon both 16.22 mg and 48.66 mg dosages (Figure 5A).

Analysis of the viral transmission within cage mates showed that mice that were not infused with SARS-CoV2-S- gamma variant pseudotyped lentivirus when left with those infused with the virus showed a slight accumulation of the virus in the nasal and the gut region. This phenomenon was seen with a high load of virus accumulation in the gut, thorax, and nasopharyngeal region among diabetic mice. While JHT and JND administration provided a slight reduction in the viral load, a combined treatment with JHT and JND for 3 days showed effective reduction in the viral load (Figure 5B). Therefore, diabetes increases the transmission of SARS-CoV2 and viral accumulation; however, combined treatment with JHT and JND reduces the susceptibility to infection.

## Discussion

Generation of variants of SARS-CoV-2 has increased the concerns about the effectiveness of drugs and even vaccines (Weisblum et al., 2020). Variants of SARS-CoV-2, like the delta and gamma mutants, show aggravated transmission and viral replication in human lung and airway tissues (Groves et al., 2021; Ostrov 2021; Plante et al., 2021; Socher et al., 2021), causing rapid onset of the immediate pulmonary effects and reduced oxygen saturation in the patients. Variants like B.1.351 have shown higher resistance to antibodies and vaccination (Kostaki et al., 2021). The potential effect of JHT will be of appreciable benefit as they are effective against all three variants.

The constituents of JHT have shown promising evidence for antiviral effects that form the rationale for the antiviral preparation. However, there is no clear efficiency of individual components demonstrated so far against SARS-CoV-2 infection. The flavonoids in *O. caudata* (Thunb.) H.Ohashi provide antiviral activity against viruses like influenza (Kwon et al., 2020). Swertisin, a flavonoid known for its antiviral activity against anti-hepatitis B and influenza virus, is also known to inhibit SARS-CoV2 RdRp in *in silico* analysis (Mahrosh and Mustafa 2021). In addition, isoliquiritigenin, an active metabolite of *G. uralensis* Fisch. Ex DC, has been previously shown to inhibit influenza A and hepatitis C in *in vitro* as well as in *in vivo* conditions. *O. japonicas* (Thunb.) Ker Gawl, consumed as a functional food in China for a long period of time, contains polysaccharides that have a cardio-protective function in conditions like diabetes (Zhang et al., 2016). The *O. japonicus* polysaccharides are a potential component of JHT in controlling comorbidities like diabetes and hypertension-associated cardiovascular disease, which are the major risk factor for SARS-CoV2 infection (de Almeida-Pititto et al., 2020). *H. cordata* Thunb is one of the components in the TCM-based formula used in China to manage the SARS-CoV outbreak (de Almeida-Pititto et al., 2020). It exhibited significant inhibitory effects on SARS- CoV 3C-like protease (3CL) and RNA-dependent RNA polymerase (RdRp). Platycodin D from *P. grandiflorum* (Jacq.) A.DC has been recently identified to prevent SARS-CoV-2 infection *via* inhibition of lysosome- and TMPRSS2-driven SARS-CoV-2-entry by disrupting the host-cell membrane cholesterol (Kim et al., 2020). Leaf extracts of *P. frutescens* (L.) Britton prevents SARS-CoV-2 viral entry into host cells by inactivating the virus and showed a synergetic improvement when treated in combination with remdesivir (Tang et al., 2021). Preclinical animal studies on *Chrysanthemum × morifolium* (Ramat.) Hemsl have proven its efficiency in treating lipopolysaccharide-induced acute lung injury in mice due to its ability to balance levels of pro-inflammatory and anti-inflammatory factors and inhibition of free radical generation (Liu et al., 2020a). Therefore, the constituents of JHT provide necessary biological factors for its effects against SARS-CoV-2 infection.

Hypertension and diabetes are seen to be the most concerning comorbidities among COVID-19 patients. Older age, hypertension, obesity, diabetes, cardiovascular disease, and chronic obstructive pulmonary disease have been commonly observed among patients with severe COVID-19 and those who did not survive. Available data from various regions of the world show that diabetes even increases the risk for COVID-19 and mortality rates (Jin and Hu 2021). In addition, diabetes reduces the normal metabolism and responses to infections, which may lead to increased viral load in diabetic individuals (Flemming 2022). In diabetic conditions, the cellular expression levels of ACE2 are normally elevated, and the metabolic switch from oxidative phosphorylation to aerobic glycolysis is established, which is correlated with the increase in viral loads (Flemming 2022).

Therefore, treatment or preventive strategies for COVID-19 infection should consider all potential complications associated with severity and mortality.

At present, conventional treatment strategies for diabetes should be decided and modified based on the severity of COVID-19 as patients treated with insulin have shown poorer outcomes than those under metformin treatment (Jin and Hu 2021). However, metformin should be discontinued in patients with respiratory troubles, acidosis, and renal and cardiac dysfunctions (Bornstein et al., 2020). DPP4 inhibitors like sitagliptin have shown beneficial effects and are considered an add-on therapy among COVID-19 patients with diabetes (Solerte et al., 2020). However, DPP4 inhibitors are known to modify the biological activities of various immunomodulatory events (Drucker 2020).

Therefore novel strategies to control COVID complications in diabetic individuals have to be identified in order to reduce the COVID mortality rates among the diabetic population. In this study, we have identified two herbal medicine-based formulations JHT and JDN that are effective against SARS-CoV-2 viral entry and accumulation. Moreover, combined treatment rapidly suppressed the accumulation of delta and gamma variants of SARS-CoV-2 pseudo viral lentivirus particles in the thorax and in the abdomen. Furthermore, the combination of JHT and JND showed suppression in the viral transmission from the infected to the uninfected SKH-1 mice. Therefore, combined administration of oral JHT and nasal JND is potentially a superior treatment strategy to prevent SARS-CoV-2 infection of various variants.

Conclusion: Various antiviral drugs to treat COVID-19 have so far shown mixed outcomes, and the generation of different variants has caused concerns about the treatment efficiency. In our study, JHT and JND have shown effective inhibition of viral entry and accumulation among various variants, which would greatly benefit in containing the spread of such variants particularly among the susceptible population with comorbidities like diabetes.

## Data Availability

The original contributions presented in the study are included in the article/Supplementary Material; further inquiries can be directed to the corresponding authors.

## Supplementary material

Supplementary Figure S1 LC-MS analysis of the JND plant extract. **(A)** Total ion (+ve) chromatogram. **(B)** Total ion (-ve) chromatogram. **(C**–**I)** Spectrum (+ve) of the selected chromatogram fraction peaks at 8.70, 10.03, 11.44, 12.01, and 17.18.

Supplementary Figure S2 LC-MS analysis of the JHT plant extract. **(A)** Total ion (+ve) chromatogram. **(B)** Total ion (-ve) chromatogram. **(C**–**E)** Spectrum (+ve) of the selected chromatogram fraction peaks at 12.05, 13.06, and 17.32.

Supplementary Figure S3 LC-MS for identifying quantification of swertisin **(A)**, isoliquiritigenin **(B),** and eupatilin **(C)** in JHT.

Supplementary Figure S4 LC-MS for identifying quantification of swertisin **(A)**, isoliquiritigenin **(B),** and eupatilin **(C)** in JND.

## Abbreviations

JHT, Jin Si Herbal Tea; JND, Jin Si nasal drop; STZ, streptozotocin; ACE2, angiotensin-converting enzyme 2; DPP4, dipeptidyl peptidase 4; APN, aminopeptidase-N; S protein, spike protein; MOI, multiplicity of infection; IVIS, *In Vivo* imaging system; 3CL, 3C-like protease; RdRp, RNA-dependent RNA polymerase.
