# Supplementary figures and images for: Combined effect of traditional Chinese herbal-based formulations Jing Si herbal tea and Jing Si nasal drop inhibits adhesion and transmission of SARS-CoV2 in diabetic SKH-1 mice
Source: Front Pharmacol. 2022 Nov 8;13:953438. doi: 10.3389/fphar.2022.953438 (PMC9681529; doi:10.3389/fphar.2022.953438)

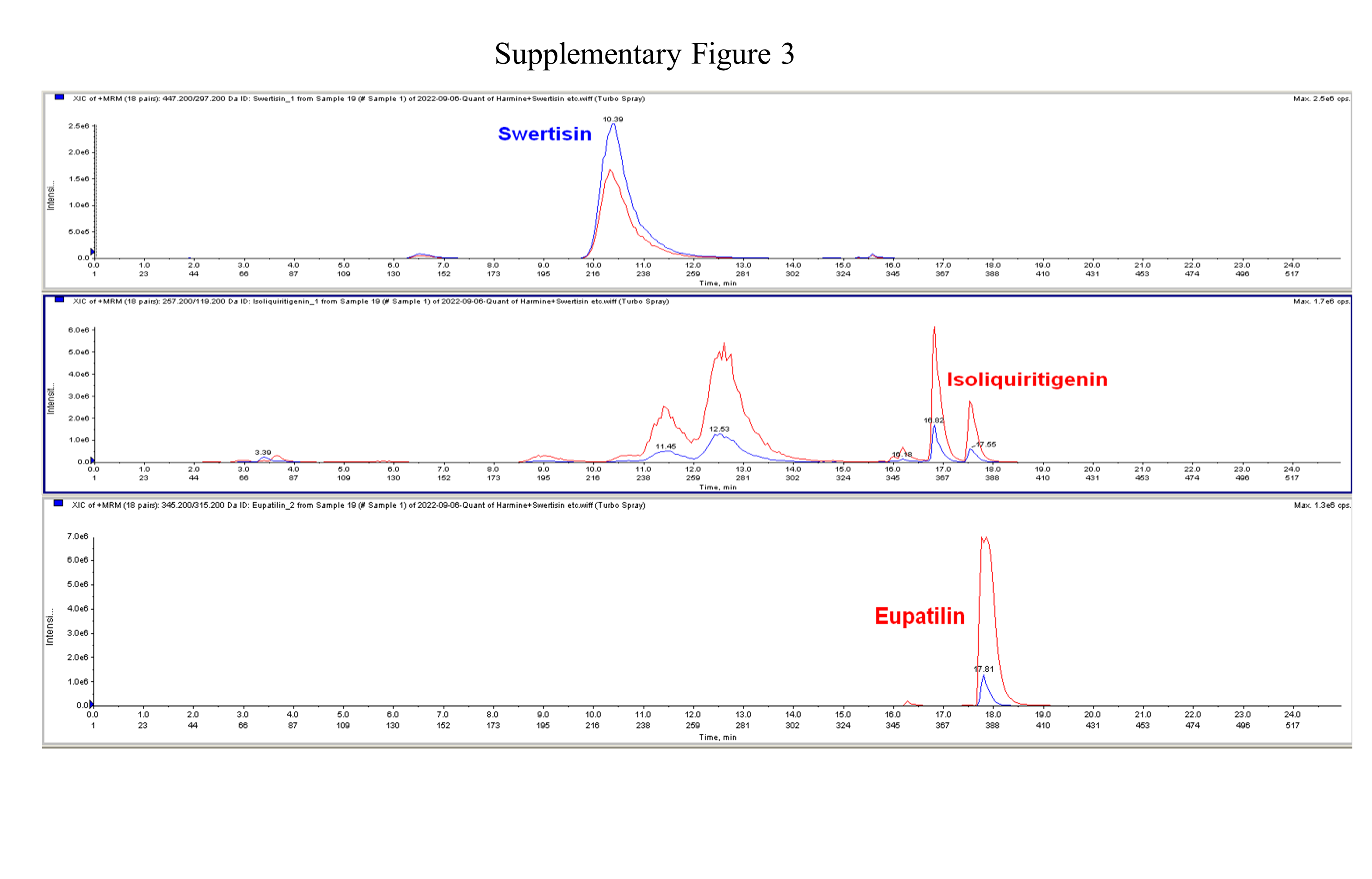

Supplement: Supplementary file 1 [file Image3.TIF]

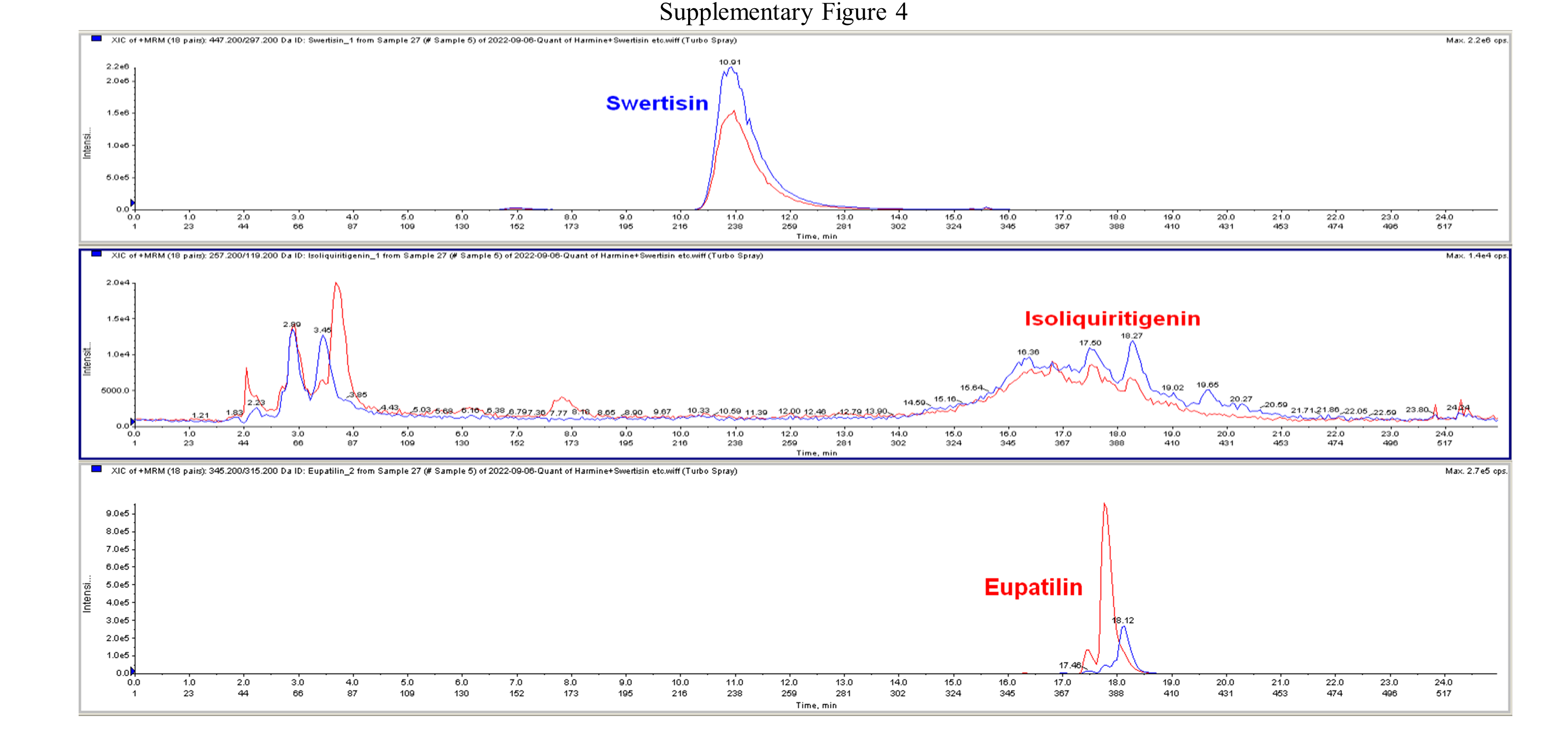

Supplement: Supplementary file 2 [file Image4.TIF]

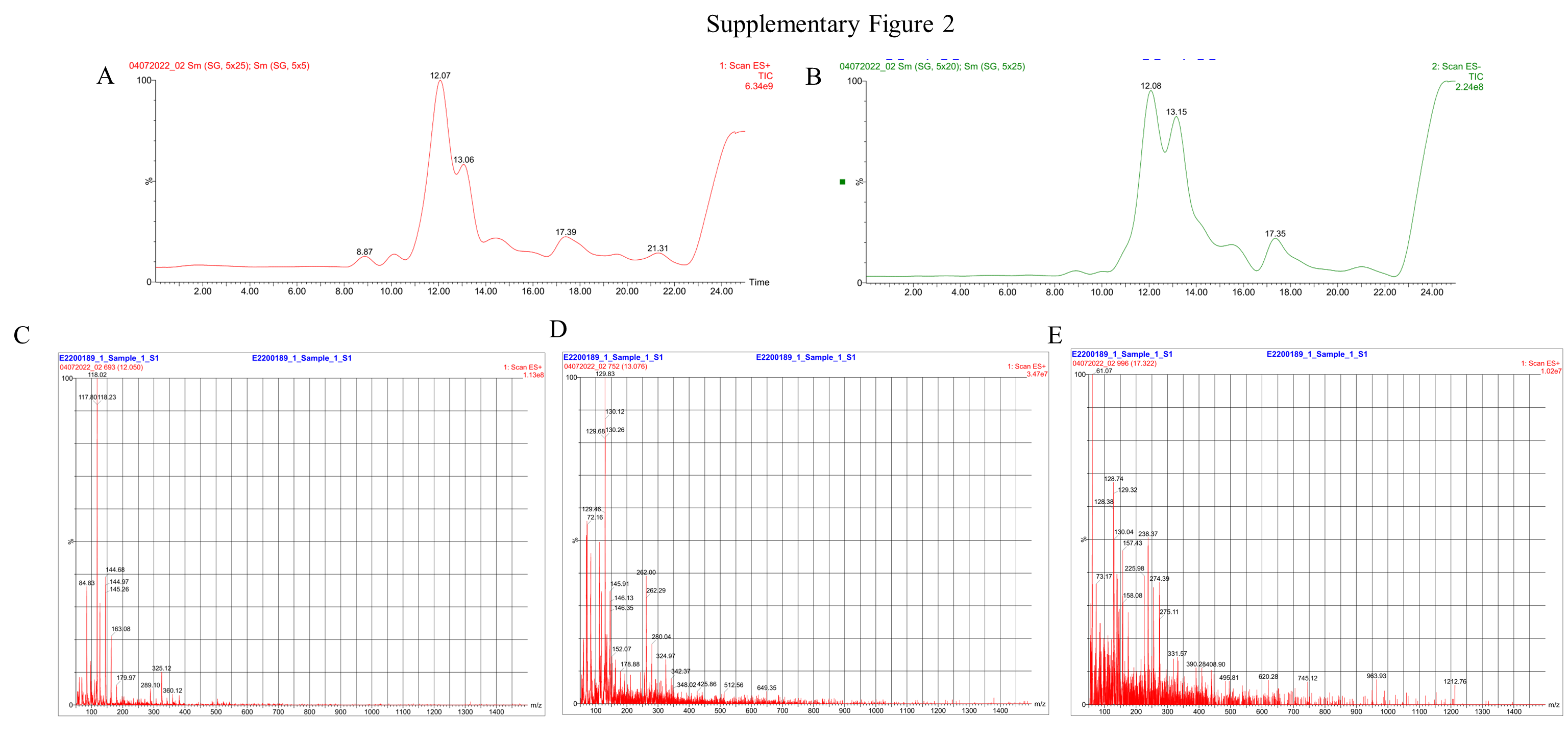

Supplement: Supplementary file 3 [file Image2.TIF]

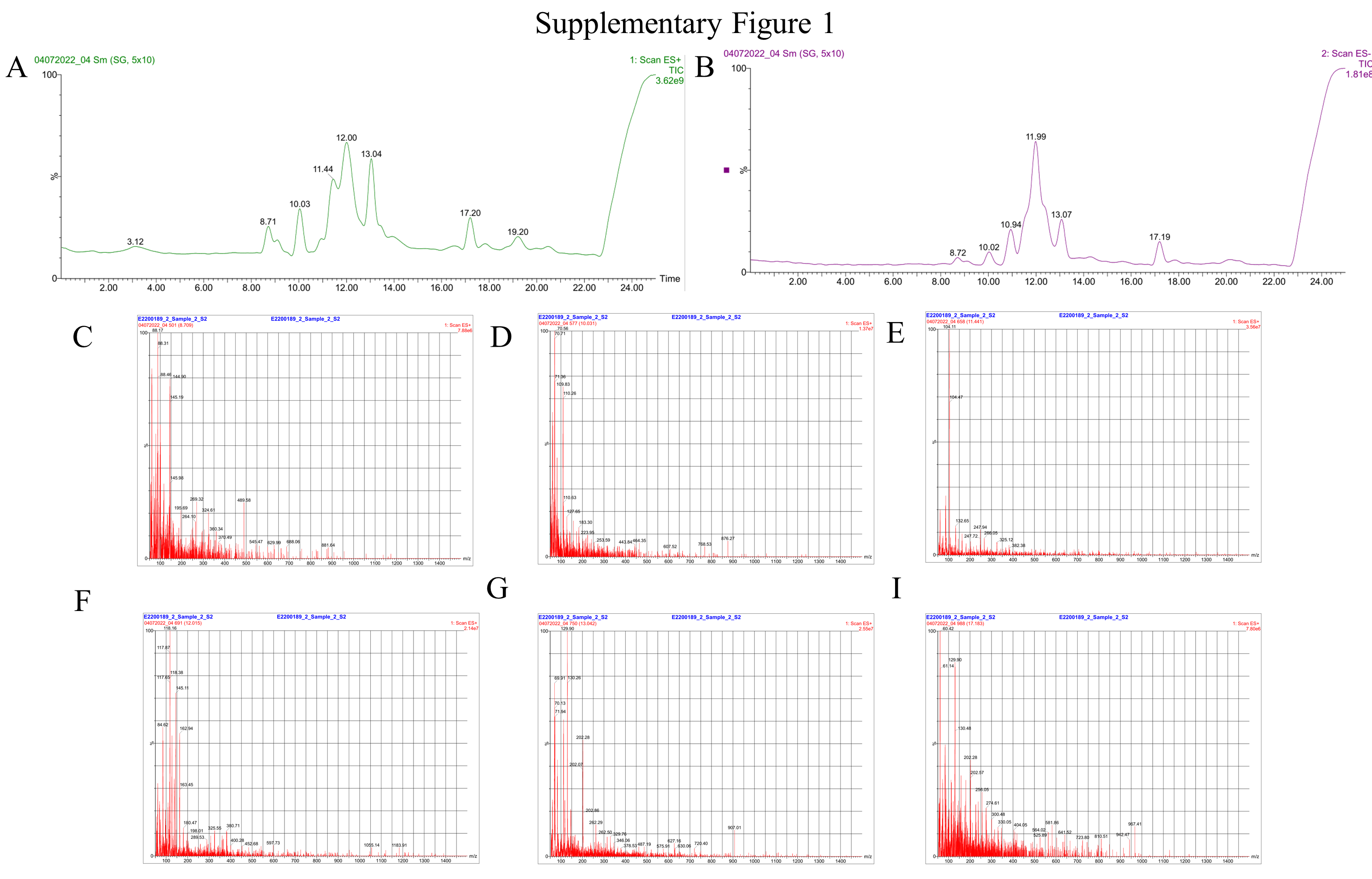

Supplement: Supplementary file 4 [file Image1.TIF]
